# A retrospective, school-based, within-class quasi-experimental evaluation of a rhythm–coordination rope-skipping programme and 1-min rope-skipping performance in primary school children

**DOI:** 10.3389/fpubh.2026.1899438

**Published:** 2026-09-10

**Authors:** Yan Bu, Junhui Zhu, Mengzhuo Yang, Yueqiu Li

**Affiliations:** 1 College of Physical Education and Health Science, Yibin University, Yibin, Sichuan, China; 2 Department of Marine Sports, Pukyong National University, Busan, Republic of Korea; 3 Faculty of Education, Universiti Kebangsaan Malaysia, Bangi, Selangor, Malaysia; 4 College of Sports and Great Health, Sichuan University of Business and Technology, Chengdu, Sichuan, China

**Keywords:** children, class clustering, constraint-led approach, nonlinear pedagogy, physical education, quasi-experimental design, rope skipping

## Abstract

One-minute rope skipping is a task-specific school performance measure influenced by physical capacity, temporal coordination, rhythm, and task familiarity; whether reorganising routine practice around rhythm- and coordination-oriented, constraint-led tasks yields additional improvement remains unclear. This retrospective, school-based, within-class, non-randomised quasi-experimental evaluation used routine records from one primary school in the Tianfu New Area, Sichuan, China, to examine whether an 8-week rhythm–coordination rope-skipping programme was associated with additional improvement in task-specific 1-min rope-skipping performance relative to usual practice. The analysis included 1,156 children nested within 30 home classes across Grades 2–6; each class had been divided non-randomly into two approximately balanced instructional subgroups using the existing roster and routine timetabling arrangements, so both conditions were represented in every class. The intervention was scheduled three times per week in 30-min sessions over 8 weeks (24 planned sessions), while the comparator continued routine physical education and usual rope-skipping practice. The primary analysis used ANCOVA with baseline adjustment, home-class fixed effects, and small-sample-corrected (CR2) standard errors clustered by class, with Satterthwaite degrees of freedom; sensitivity analyses included class random-intercept, change-score, class-level, wild-cluster-bootstrap, and leave-one-class-out specifications. Groups were comparable at baseline (all standardised differences ≤ 0.05). Mean performance increased from 120.4 to 128.8 skips in the intervention group and from 118.5 to 121.9 skips in the comparator group. In the primary class-adjusted analysis, the intervention was associated with an additional difference of 6.36 skips (95% CI 3.51 to 9.21; *p* < 0.001; standardised adjusted difference 0.17). No validated minimal important difference has been established for this outcome, so the educational importance of the 6.36-skip difference remains uncertain despite its statistical detectability. Alternative class-aware sensitivity models yielded estimates ranging from 5.32 to 6.42 skips, while leave-one-class-out estimates ranged from 5.87 to 6.92 skips across 30 refitted models; all estimates were directionally consistent. Participation in the rhythm–coordination programme was associated with a small additional improvement in task-specific 1-min rope-skipping performance after accounting for class-level clustering; the findings do not establish a causal mechanism or demonstrate transfer to broader motor competence, and prospective multi-school studies with documented allocation, fidelity monitoring, blinded assessment, and broader motor outcomes are required.

## Introduction

Rope skipping is one of the most accessible activities in school physical education. It requires minimal equipment and space, scales easily to large classes, and is embedded in physical-education curricula, after-school programmes, and campus fitness testing in many countries. Systematic reviews indicate that jump-rope training can improve health- and sport-related fitness in children and adolescents, although the magnitude of benefit depends on participants’ age, training frequency, and programme content (1, 2). As a result, the 1-min rope-skipping test is commonly used as a task-specific measure of school rope-skipping performance. More broadly, school physical education is intended to support physical competence, neuromuscular development, mental wellbeing, academic focus, and social development, underscoring that a task-specific performance count should not be treated as a proxy for broader physical or psychosocial development (3).

Successful continuous rope skipping requires repeated coordination of rope rotation, take-off, landing, and postural adjustment within a temporal sequence. Accordingly, task performance may be influenced by both physical capacity and coordinative demands. However, the relative contributions of rhythm, coordination, endurance, and task familiarity cannot be determined from the 1-min count alone. Consistent with a coordinative component to the task, rope-skipping practice has been linked not only to fitness but also to motor-coordination and attentional outcomes in children (4, 5), and to broader health benefits when delivered as structured training (6). This suggests that how rope skipping is taught—not only how much children practise—may shape performance gains on this task-specific measure.

In everyday school practice, rope-skipping instruction is often organised around repetition and counting: children are encouraged to “jump more” or “jump faster” and are assessed mainly on total counts. Repetition and count-focused practice is simple to organise, but it may provide limited information about how children regulate timing, recover from rope interruptions, and adapt their movement pattern during continuous skipping.

The constraint-led approach and nonlinear pedagogy offer an alternative framework. Rather than assuming that all children should converge on a single ideal technique through direct instruction, this perspective treats motor learning as an adaptive search for functional movement solutions under interacting task, environmental, and individual constraints (7). By manipulating task goals, informational cues, and practice boundaries—for example, segmenting continuous skipping, providing rhythmic cues, limiting errors, or pairing children to synchronise their rhythm—this approach is intended to guide learners toward more stable task execution (8, 9). Such approaches have been associated with improvements in movement competence and related cognitive outcomes in primary-school physical education (8, 10). In this context, rhythmic cueing may provide a temporal reference, segmentation may reduce task complexity during skill acquisition, error-recovery practice may help children resume coordinated action after interruptions, and peer synchronisation may provide an external pacing reference. These features provide a theoretical reason to expect greater task-specific improvement than from count-focused repetition alone, without implying that these mechanisms were directly measured in the present evaluation.

Despite this theoretical promise, evidence on how rhythm- and coordination-oriented tasks can be embedded within routine physical education remains limited. Most school-based motor-learning and fundamental-movement-skill interventions are delivered as add-on or specialist programmes, and reviews continue to call for pragmatic, context-sensitive interventions that can be sustained within existing curricula and large class sizes (11, 12). Rhythmic physical-activity programmes have shown benefits for gross-motor outcomes in young children (13), but few studies have tested whether reorganising the everyday rope-skipping that schools already deliver, around constraint-led principles, produces measurable additional gains in the standard 1-min test under real-world conditions.

This evaluation used routinely collected school records, subsequently linked to home-class identifiers, to estimate whether an 8-week, pragmatic, constraint-led rhythm–coordination rope-skipping programme delivered within regular physical-education classes in a single primary school was associated with additional improvement in task-specific 1-min rope-skipping performance relative to usual school practice. The primary aim was to estimate this association after accounting for the school’s existing home-class structure. Because the evaluation was non-randomised and based on routinely collected school records, the study did not aim to establish a causal mechanism or general motor-learning transfer. Accordingly, the directional hypothesis tested in this retrospective evaluation was that, after adjustment for baseline performance and home-class structure, participation in the rhythm–coordination condition would be associated with higher post-programme 1-min rope-skipping performance than usual practice.

## Materials and methods

### Study design and reporting

This was a retrospective, two-group, pretest–posttest, school-based, within-class quasi-experimental evaluation conducted in one primary school in the Tianfu New Area, Sichuan Province, China, using routinely collected physical-education and school fitness records. The 1,156 children were nested within 30 home classes across five actual school-grade cohorts, spanning Grades 2 to 6 (Grade 6: 4 classes, *n* = 149; Grade 5: 6 classes, *n* = 216; Grade 4: 6 classes, *n* = 242; Grade 3: 6 classes, *n* = 254; Grade 2: 8 classes, *n* = 295). School-recorded grade and class membership were used in preference to grade estimates derived from date of birth; an earlier date-of-birth-derived cohort variable, which had incorrectly separated a small number of children from their actual enrolled grade, was discarded once school enrolment records became available. Both intervention and comparator participants were represented within each home class. Group assignment was non-random. The study is reported with reference to the TREND statement for non-randomised evaluations (14), supplemented by relevant cluster-reporting principles and the TIDieR checklist for intervention description (15).

### Ethics and consent

The rhythm–coordination programme and the associated baseline and post-programme assessments were implemented as part of the school’s routine physical-education provision and routine educational monitoring. Baseline testing was conducted on 6 March 2026, and the programme was delivered over eight teaching weeks from 9 March to 1 May 2026; post-programme testing was conducted on 4 May 2026.

Ethical approval for the secondary research use, analysis, and dissemination of the de-identified school records was granted by the Science and Technology Ethics Committee of Yibin University on 21 April 2026 (approval No. YBULLKY26042101). The research analysis introduced no additional testing, invasive procedures, or participant contact beyond the routine educational activities and assessments already undertaken. Yibin University served as the research-governance institution for the academic analysis and dissemination of the school-based records, while the participating school provided the educational setting and authorised access to the records.

Written informed consent for the secondary research use of the children’s school records was obtained from the parents or legal guardians of all participants, and age-appropriate assent was obtained from the children.

### Setting, participants, and analytic sample

Children were eligible to participate in the routine school programme if they were enrolled in a participating class and had no medical restriction precluding participation in regular physical education. For the secondary research analysis, records were included when valid baseline and post-programme 1-min rope-skipping scores, study-condition information, sex, and home-class identifiers were available and could be reliably linked.

The archived analytic source files contained 1,156 children with complete paired records nested within 30 home classes. Counts of children who were absent, medically exempted, or lacked paired records before creation of the archived files were not retained in the research dataset. Age was derived from each child’s date of birth at the baseline reference date.

### Assignment and instructional organisation

Participants were not individually randomised. Before baseline scores were accessed for research analysis, the school’s physical-education administration divided each home class into two approximately balanced instructional subgroups using the existing class roster and routine timetabling arrangements. The archived records do not retain a more granular administrative allocation rule (for example, alternating roster order or teacher-by-teacher assignment), so the exact operational sequence of subgroup formation cannot be reconstructed. The research team did not reassign children after baseline testing, and subgroup allocation was not based on rope-skipping performance. The archived records also do not permit retrospective verification of whether teachers knew the intended condition at the time the administrative subgrouping was formed. During the relevant physical-education periods, the two subgroups completed their assigned activities in separate activity stations or instructional rotations under the supervision of the school’s physical-education teachers. The available records do not permit reliable reconstruction of whether the same teacher supervised both conditions in every class or lesson, whether both conditions were delivered concurrently within the same physical-education lesson period on every occasion, or whether station-specific environmental conditions were equivalent; these factors were therefore retained as potential sources of instructional confounding. Participants remained members of the same home classes; therefore, informal exchange of practice strategies between conditions could not be completely excluded.

### Intervention

The intervention group completed a rhythm–coordination rope-skipping programme delivered three times per week in 30-min sessions within regular physical-education lessons over 8 weeks (24 planned sessions; planned total dose 720 min). The programme operationalised the constraint-led approach by systematically manipulating task, environmental, and individual constraints rather than emphasising repetition and counting. Task difficulty was progressed through bout duration, tempo, error tolerance, and coordination demands rather than through a prescribed physiological intensity (see Tables 1, 2).

**Table 1:** Structure of each 30-min intervention session.

Session phase	Duration	Content
Warm-up	5 min	Joint mobility, low-intensity bouncing, rope-length adjustment, basic rhythm activation
Segmented continuous skipping	7 min	Progressive extension from short, achievable bouts toward sustained continuous skipping
Rhythmic cueing tasks	7 min	Teacher verbal cues, clapping, and visual or auditory rhythm cues
Error recovery and peer synchronisation	6 min	Reducing trip-ups, rapid recovery after interruption, peer tempo matching
Integrated practice	3 min	Continuous skipping at an individually stable rhythm
Cool-down and feedback	2 min	Relaxation and brief teacher feedback

**Table 2:** Eight-week progression of the intervention programme.

Weeks	Primary focus
1–2	Rope control, stable take-off, basic rhythm
3–4	Segmented continuous skipping, reduced trip-ups, rhythm recovery
5–6	Rhythm variation, peer synchronisation, increased coordination demands
7–8	Extended continuous duration, task integration, approaching stable 1-min performance

An error was defined as interruption of rope rotation, failure of the rope to pass beneath the feet, loss of the intended temporal sequence, or the need to restart the movement. Children were paired approximately by body size and current task proficiency. Each child used an individual rope, and partners attempted to match tempo while remaining spatially separated; the leading partner was alternated across repetitions. Sessions were delivered by the school’s physical-education teachers using equipment and facilities already available at the school. Across the planned programme, the six session phases and the overall 30-min structure were core components scheduled for each session. Within those phases, task demands could vary through bout duration, tempo, error tolerance, cueing, and peer matching according to current task proficiency; these adaptations altered task difficulty rather than the core phase sequence.

### Programme fidelity and received dose

Programme implementation was retrospectively verified using the school timetable, available lesson plans, teaching-progress records, and structured confirmation from the physical-education teachers. These sources confirmed delivery across the planned 8-week period and the use of the core intervention components. Because individual session-level attendance and a prospective quantitative fidelity checklist were not retained, the exact received dose at the individual-child level could not be calculated. The intervention was scheduled for 24 sessions; available records confirmed delivery across the 8-week period but did not permit exact reconstruction of every session for every child.

### Control condition

The comparator group continued routine physical education and the school’s usual rope-skipping activities during the same 8-week period. Usual practice consisted primarily of self-paced continuous skipping, repetition of the standard movement, count-focused practice, and general teacher feedback. It did not systematically incorporate the structured rhythmic cueing, segmented progression, error-recovery goals, tempo variation, or peer-synchronisation tasks used in the intervention condition. The comparison therefore estimates the added value of reorganising usual rope-skipping practice rather than the effect of rope skipping compared with no rope skipping. Out-of-class rope-skipping practice was not systematically recorded and could not be included as a covariate. Routine records did not quantify session-by-session rope-skipping minutes or total practice volume in the comparator subgroup, so exposure could not be dose-matched or entered as a covariate. The estimated between-condition association may therefore reflect differences in total exposure as well as differences in instructional organisation.

### Outcome assessment

The primary and only outcome was task-specific 1-min rope-skipping performance, defined as the number of valid skips completed within 60 s. Children received the same standardised instructions at both assessments. Rope length was adjusted to the child before testing. One valid skip was counted when the rope passed completely beneath the feet and the child completed the corresponding jump. Following a rope interruption, the child was allowed to resume immediately; only successfully completed skips were counted. The same test duration, scoring definition, equipment requirements, and school testing procedures were used at baseline and post-programme assessment.

Formal allocation blinding was not feasible in the routine school setting. However, assessors followed a standardised counting protocol and did not consult the child’s baseline score while scoring the post-programme assessment. No study-specific test–retest or inter-rater reliability coefficient was available. Measurement consistency was supported through standardised timing, scoring definitions, instructions, and testing procedures, but the absence of a study-specific reliability estimate was retained as a limitation. The archived records did not preserve assessor identities, a formal assessor-training log, or information sufficient to confirm whether the same assessors tested each child at both time points. These details could not be reconstructed retrospectively.

### Data linkage and privacy

Baseline and post-programme records were linked within the authorised school data environment using home-class membership, name, sex, and date of birth. Class membership was used to distinguish children with otherwise identical demographic combinations. After linkage and verification, names and full dates of birth were removed and replaced with pseudonymous student and class identifiers before statistical analysis.

### Statistical analysis

Continuous variables are summarised as mean ± standard deviation (and median where distributions were skewed) and categorical variables as counts and percentages. Baseline comparability between conditions was assessed primarily using standardised differences, with Welch’s t tests and chi-square tests reported for descriptive purposes only; standardised differences below 0.10 were regarded as negligible imbalance.

The primary analysis used ANCOVA with the post-programme score as the dependent variable, study condition as the exposure, baseline score and sex as individual-level covariates, and home-class fixed effects. Standard errors were clustered by home class and corrected for the limited number of clusters using the CR2 bias-reduced-linearisation cluster-robust variance estimator with Satterthwaite degrees of freedom (16). Home-class fixed effects were used because every class contributed children to both study conditions, which allows the class term to absorb shared grade level, teacher, class climate, peer environment, lesson timing, and other class-level characteristics without discarding within-class contrasts. CR2/Satterthwaite inference was selected because conventional cluster-robust standard errors can be downward biased when the number of clusters is modest; CR2 provides small-sample bias reduction together with Satterthwaite-adjusted degrees of freedom (16). Home class, rather than the condition-specific instructional subgroup, was used as the clustering unit because the two subgroups shared the same class context and could not reasonably be assumed independent. Clustering at the broader home-class level allows arbitrary residual dependence among children within either subgroup and across the two subgroups of the same class, including dependence arising from shared teachers, peer environment, lesson timing, and possible contamination. Treating condition-specific subgroups as separate clusters would instead impose independence between the two subgroups from the same home class; this was therefore not used as the primary variance structure.

Age and school grade were not entered simultaneously in the primary model because of their near-redundancy with each other and with the home-class fixed effects, which already account for grade and other shared class-level characteristics. ANCOVA was selected because it estimates the between-condition post-programme difference conditional on baseline performance and is generally the more efficient and less biased approach when baseline and follow-up measurements are both available (17, 18); however, because assignment was non-random, baseline adjustment cannot eliminate unmeasured confounding, and change-score and class-level analyses were therefore used to assess sensitivity to model specification. The homogeneity-of-regression-slopes assumption was assessed by adding a condition-by-baseline interaction term to the primary model.

Sensitivity analyses comprised: (i) a class random-intercept linear mixed-effects model; (ii) a class-fixed-effects change-score model; (iii) an analysis of the 30 class-level mean differences between conditions; (iv) a wild cluster bootstrap using Webb six-point weights with 9,999 replications under the restricted (null-imposed) data-generating process (19, 20); (v) a leave-one-class-out analysis; and (vi) exclusion of extreme change scores (> 3 SD from the mean change). The standardised adjusted difference was calculated by dividing the class-adjusted coefficient by the pooled baseline standard deviation. A group-by-grade-cohort interaction was evaluated using a joint cluster-robust Wald test (CR2 variance, HTZ/Satterthwaite-type denominator degrees of freedom), with home class as the clustering unit, to examine whether the class-adjusted estimate varied across the five grade cohorts. Two-sided *p* < 0.05 was considered statistically significant. This evaluation was not prospectively registered. Data management and descriptive analyses were conducted in Python 3.12 (pandas, NumPy); the class fixed-effects, cluster-robust, and mixed-effects models were fitted in R 4.3.3 using the lme4 (version 1.1–35.1), sandwich (version 3.1–0), and clubSandwich (version 0.5.10) packages, with a fixed random-number seed (20260724). The wild cluster bootstrap was implemented using a custom base-R bootstrap-t function with Webb six-point weights; the complete implementation is provided in S2 File.

## Results

### Participant flow and sample characteristics

**Figure 1:**
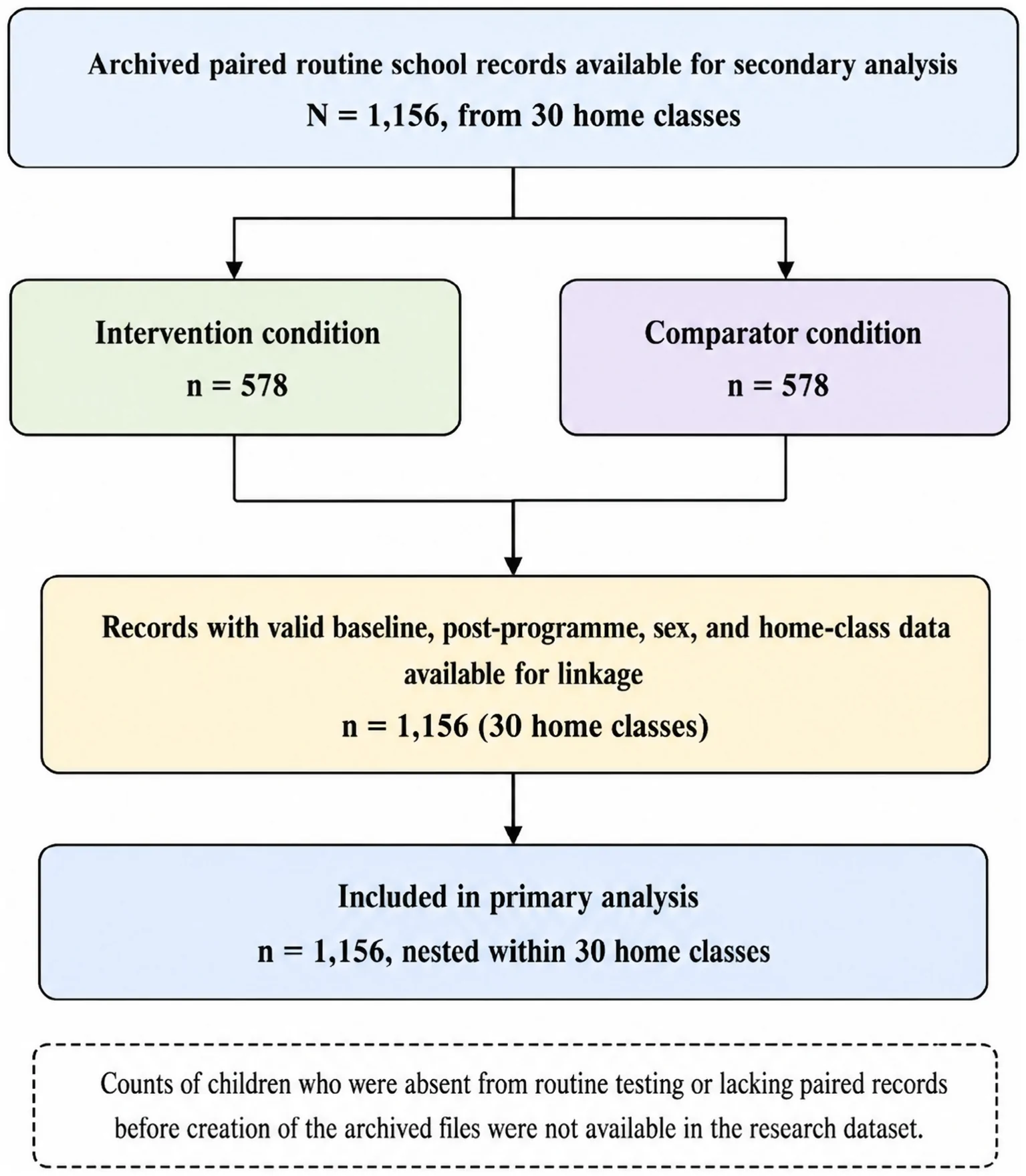
Participant flow through the retrospective secondary analysis. Both study conditions were represented within each of the 30 home classes; counts of children absent from routine testing or lacking paired records before creation of the archived files were not available in the research dataset.

The archived analytic dataset comprised 1,156 children nested within 30 home classes: 578 in the intervention condition and 578 in the comparator condition, with both conditions represented in every home class (Figure 1). All 1,156 children with valid, linkable paired records were included in the primary analysis.

### Baseline comparability and class distribution

Groups were well balanced at baseline on all measured characteristics. The standardised differences were negligible for baseline 1-min performance (0.05), age (0.05), and sex (0.04) (Table 3). Baseline rope-skipping performance was 120.4 ± 38.0 skips in the intervention group and 118.5 ± 38.1 skips in the comparator group; the standardised baseline difference was 0.05, indicating negligible imbalance.

**Table 3:** Baseline characteristics by study condition.

Characteristic	Intervention (*n* = 578)	Comparator (*n* = 578)	Std. difference
Girls, *n* (%)	285 (49.3)	273 (47.2)	0.04
Boys, *n* (%)	293 (50.7)	305 (52.8)	—
Age, years, mean ± SD	9.78 ± 1.40	9.72 ± 1.38	0.05
Baseline 1-min skips, mean ± SD	120.4 ± 38.0	118.5 ± 38.1	0.05

SD, standard deviation. Standardised differences below 0.10 indicate negligible imbalance. This table provides an individual-level baseline description; all 30 home classes contributed both conditions, and home-class structure is reported in Table 4 and incorporated in the adjusted analyses.

Thirty home classes contributed participants to both conditions, spanning five grade cohorts (Grades 2–6; Table 4). Overall class size was 38.5 ± 3.5 children (median 38; IQR 37.0–40.8; range 32–46). Within each class, the intervention subgroup averaged 19.3 ± 3.6 children (range 9–27) and the comparator subgroup averaged 19.3 ± 2.9 children (range 15–26).

**Table 4:** Number of classes and children per grade and per study condition.

Grade	Classes contributing to both conditions	Intervention, *n*	Comparator, n	Total
Grade 6	4	84	65	149
Grade 5	6	103	113	216
Grade 4	6	119	123	242
Grade 3	6	127	127	254
Grade 2	8	145	150	295
Total	30	578	578	1,156

Every class listed contributed children to both the intervention and comparator conditions; grade-level counts are therefore not independent of the class counts in Table 3’s companion text.

### Descriptive pretest–post-programme changes

**Figure 2:**
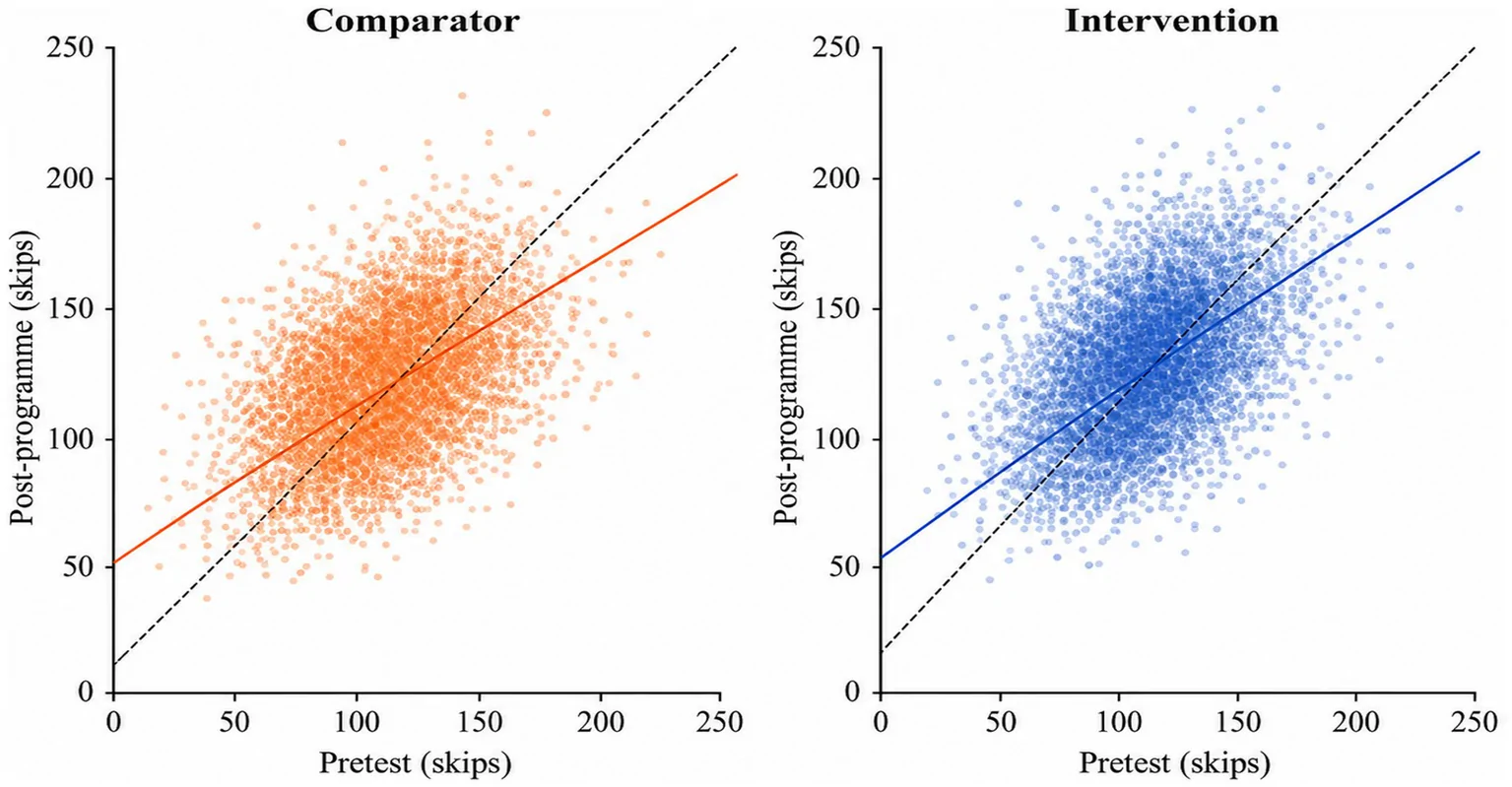
Pretest–post-programme performance by study condition (*n* = 1,156; individual children, semi-transparent points). The dashed grey line marks equality (no change); the solid coloured line is the fitted linear regression of post-programme on pretest score within each condition.

One-minute performance increased in both conditions from baseline to post-programme assessment (Table 5). The unadjusted mean change was +8.3 skips (median +7.0) in the intervention group and +3.4 skips (median +2.0) in the comparator group. These within-condition changes are reported descriptively; the primary inference is the adjusted between-condition comparison presented in Table 6, Figure 2.

**Table 5:** Pretest, post-programme, and change scores in 1-min rope-skipping performance by study condition.

Group	Pretest, mean ± SD	Post-programme, mean ± SD	Change, mean (median)
Intervention (*n* = 578)	120.4 ± 38.0	128.8 ± 31.1	+8.3 (+7.0)
Comparator (*n* = 578)	118.5 ± 38.1	121.9 ± 29.8	+3.4 (+2.0)

SD, standard deviation. Change, post-programme − pretest. These within-condition changes are descriptive only; inferential interpretation is based on the adjusted between-condition estimates in Table 6.

**Table 6:** Primary and sensitivity estimates of the between-condition difference in 1-min rope-skipping performance, accounting for home-class clustering.

Model	Estimate (skips)	95% CI	*p*
Class fixed-effects ANCOVA — primary (CR2 class-clustered, Satterthwaite df)	6.36	3.51 to 9.21	< 0.001
Class random-intercept mixed model (Wald CI)	6.16	3.24 to 9.07	< 0.001
Class-adjusted change-score model	5.66	2.57 to 8.74	< 0.001
30-class-level average difference	5.32	2.13 to 8.51	0.002
Wild cluster bootstrap (Webb 6-point, B = 9,999)	6.36	3.48 to 9.24	0.0001
Leave-one-class-out (range across 30 refits)	5.87 to 6.92	—	—
Excluding extreme change scores (> 3 SD, n = 10)	6.42	3.63 to 9.21	< 0.001
Condition × baseline interaction	0.011 (β)	−0.10 to 0.13	0.845

CI, confidence interval. Positive estimates favour the intervention. Standardised adjusted difference = 6.36 ÷ 38.08 (pooled baseline SD) = 0.17. The wild-cluster-bootstrap p-value reflects the minimum reportable value with *B* = 9,999 replications (add-one correction: 1/(*B* + 1) = 0.0001). The condition × baseline interaction row reports the interaction coefficient, not a between-condition difference, and tests homogeneity of regression slopes rather than the primary effect. The primary ANCOVA coefficient is an individual-level adjusted post-programme contrast; the 30-class estimate is the mean of class-specific between-condition differences in change; and the leave-one-class-out entry is the range of the primary coefficient across 30 refits, not a confidence interval.

### Primary class-adjusted analysis

In the primary class fixed-effects ANCOVA with CR2 class-clustered, small-sample-corrected standard errors, participation in the intervention condition was associated with an adjusted post-programme difference of 6.36 skips (SE 1.39; Satterthwaite df 28.7; 95% CI 3.51 to 9.21; *p* < 0.001; model *R*^2^ = 0.346; standardised adjusted difference = 0.17). Across the alternative class-aware sensitivity models that produced a single overall estimate, estimates ranged from 5.32 to 6.42 skips. In the leave-one-class-out analysis, estimates ranged from 5.87 to 6.92 skips across the 30 refitted models; all estimates were directionally consistent with the primary result (Table 6). There was no evidence that the baseline–post-programme association differed by condition (condition × baseline: β = 0.011, SE 0.056, df 24.4, 95% CI − 0.10 to 0.13; *p* = 0.845), supporting the homogeneity-of-regression-slopes assumption underlying the primary ANCOVA.

### Class-level heterogeneity

**Figure 3:**
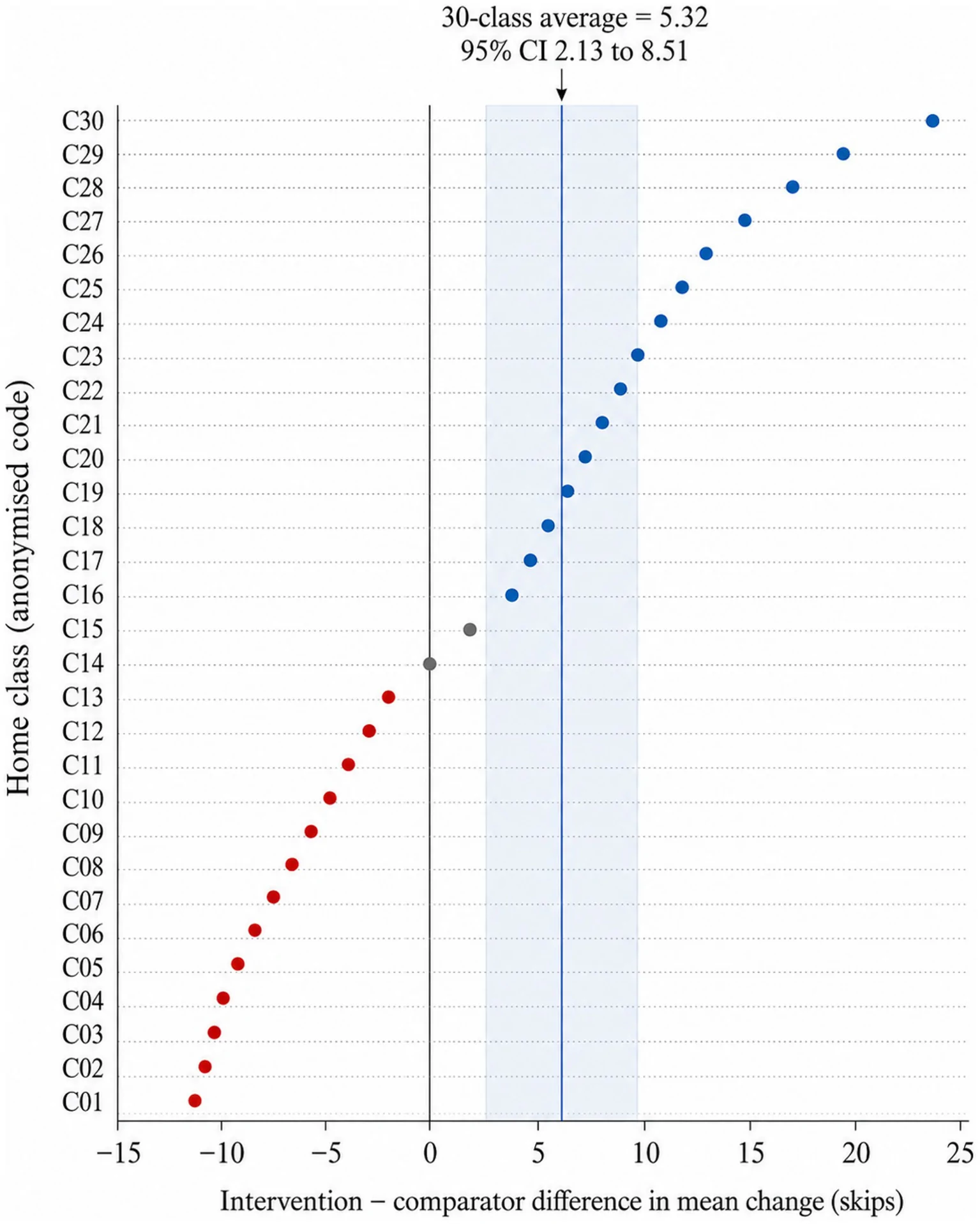
Class-specific intervention–comparator differences in mean change across the 30 home classes (one point per home class, sorted by magnitude; classes are identified by an anonymised code and are not the school’s internal class numbers). The vertical line and shaded band show the 30-class average difference and its 95% CI (5.32; 2.13 to 8.51). This class-level descriptive estimate is distinct from the individual-level adjusted ANCOVA coefficient (6.36 skips) and the leave-one-class-out range (5.87 to 6.92) reported in Table 6.

The magnitude of the within-class intervention–comparator difference varied across classes. Twenty classes showed a difference greater than 2 skips favouring the intervention, two classes showed a difference within ±2 skips of zero, and eight classes showed a difference greater than 2 skips favouring the comparator; per-class differences ranged from −11.9 to +22.9 skips (median +6.5) (Figure 3). The average class-level difference favoured the intervention, consistent with the primary model. Because teacher identity by subgroup, session-level fidelity, and exact subgroup exposure were not available, the observed between-class spread could not be attributed to a specific source.

### Sensitivity analyses and data re-audit

The class-adjusted estimate was materially unchanged when extreme change scores (> 3 SD, *n* = 10) were excluded (6.42 skips, 95% CI 3.63 to 9.21; Table 6). The wild cluster bootstrap (*p* = 0.0001) and the leave-one-class-out analysis (estimates ranging from 5.87 to 6.92 across the 30 refits) were consistent with the primary estimate, as was the group-by-grade-cohort joint cluster-robust Wald test (*F*(4,11) = 0.48; *p* = 0.748), which found no evidence that the class-adjusted difference varied across the five grade cohorts. Grade-cohort-stratified point estimates, together with the corresponding class-adjusted confidence intervals, are reported in S2 Table rather than in the main text, because the number of classes within some individual cohorts (4 to 8) is too small for the Satterthwaite approximation to be fully reliable at the cohort level.

The standard deviation decreased in both groups from baseline to post-programme assessment (intervention: SD 38.0 to 31.1; comparator: SD 38.1 to 29.8). Re-audit of the class-identified records found no fully duplicated rows (matching on class, sex, date of birth, pretest, and post-programme score) and no obvious ceiling or floor clustering in the score distributions (pretest range 8–233; post-programme range 15–208). The symmetric reduction may reflect regression toward the mean, greater improvement among initially lower-performing children, task familiarisation, or measurement variability; the study could not distinguish among these explanations.

## Discussion

In this retrospective school-based within-class quasi-experimental evaluation, participation in the rhythm–coordination programme was associated with a small additional improvement in task-specific 1-min rope-skipping performance after accounting for shared home-class characteristics and class-level clustering. The primary class fixed-effects ANCOVA estimated an adjusted difference of 6.36 skips (95% CI 3.51 to 9.21). Alternative class-aware sensitivity models yielded estimates ranging from 5.32 to 6.42 skips, while leave-one-class-out estimates ranged from 5.87 to 6.92 skips across the 30 refitted models. Although the direction of the estimates was consistent, the magnitude of the within-class difference varied across classes.

The between-class spread should not be interpreted as evidence of uniform implementation or uniform response. Plausible contributors include differences in teacher delivery, implementation fidelity, class composition, peer dynamics, local instructional conditions, and sampling variability, but the available retrospective records do not permit these explanations to be separated. The consistency of the class-aware sensitivity analyses therefore supports robustness of the observed association to model specification, not elimination of unmeasured confounding from the non-random allocation mechanism.

The observed pattern is compatible with the hypothesis that rhythmic cueing, segmented practice, and error-recovery tasks may support more stable execution of the rope-skipping task. This interpretation is consistent with the constraint-led approach and nonlinear pedagogy, in which learners are guided toward individually adaptable coordination solutions rather than a single prescribed technique (7–9), and with evidence that rhythm- and coordination-oriented activities can benefit children’s motor outcomes (4, 13). However, rhythm stability, inter-limb coordination, movement quality, and performance on untrained motor tasks were not measured. The study therefore cannot distinguish broader motor-learning transfer from task-specific practice, familiarisation, or differences in total exposure.

The estimated difference (6.36 skips) corresponded to approximately 5% of the post-programme group mean (121.9–128.8 skips) and represented a small standardised effect (0.17 pooled-baseline-SD units). No validated minimal important difference was identified for this school-based 1-min rope-skipping outcome. The result should therefore be interpreted as a modest task-specific gain rather than evidence that a recognised educationally meaningful threshold was achieved.

From a practical standpoint, the programme used equipment the school already owned, was delivered by the school’s own physical-education teachers, required no external specialist personnel, and was embedded within normal physical-education lessons. Because the comparator group also continued rope skipping as usual, the comparison reflects the added value of reorganising existing rope-skipping instruction rather than rope skipping versus no rope skipping. These features are relevant to the programme’s practicality within routine school settings, but they do not, on their own, establish that the approach generalises beyond the single school studied here.

The direction and size of the present results are broadly consistent with the wider literature on school-based motor and fundamental-movement-skill interventions, which typically reports small-to-moderate benefits that depend heavily on dose, content, and delivery context (11, 12, 21–23). They also align with quasi-experimental work on functional and skill-oriented physical-education programmes (24, 25). We are cautious not to over-interpret the comparison, however, because differences in populations, outcomes, and intervention intensity make direct numerical comparison difficult.

### Limitations

This evaluation has several limitations. Group assignment was non-randomised, and unmeasured selection bias may therefore remain. Potential differences in motivation, prior rope-skipping experience, teacher expectations, or peer dynamics may have differed between subgroups and influenced subsequent performance despite negligible imbalance in measured baseline characteristics. The exact operational allocation rule, teacher condition awareness at subgroup formation, whether the same teacher supervised both conditions in every class or lesson, and station-specific environmental equivalence could not be fully reconstructed from the archived records. Because both study conditions were drawn from the same home classes, informal contamination between conditions could not be completely excluded; exchange of strategies could have reduced the contrast between conditions and attenuated the observed between-condition difference, although its direction and magnitude cannot be quantified. Individual session-by-session attendance was unavailable, the exact dose received by each child could not be quantified, and the total volume of practice completed by the comparator group was not measured. Out-of-class rope-skipping practice was also not recorded. Formal outcome-assessor blinding was not implemented, assessor identities and a formal training log were not retained, and no study-specific reliability coefficient was available for the outcome measure. In addition, only a single, task-specific performance outcome was assessed, and the evaluation was not prospectively registered. Records of initial absence and attrition before creation of the archived analytic files may have been incomplete. Finally, the evaluation was conducted in a single school, and, as a retrospective secondary analysis, it cannot establish a causal mechanism.

Future research should use cluster-randomised controlled designs with the class or instructional subgroup as the unit of allocation, with analysis aligned to the allocation unit, accompanied by structured, prospective fidelity monitoring (attendance, dose, and protocol adherence), formal outcome-assessor blinding, and measurement of out-of-class activity. Incorporating direct, process-oriented measures—such as rhythm stability, inter-limb coordination, error frequency, movement variability, and broader motor competence—alongside the 1-min count would help test the proposed mechanisms and determine whether constraint-led rope skipping confers transferable motor-learning benefits. Multi-school samples and longer follow-up would clarify generalisability and the durability of gains.

## Conclusion

In this retrospective school-based within-class quasi-experimental evaluation, participation in the rhythm–coordination programme was associated with a small additional improvement in task-specific 1-min rope-skipping performance after accounting for class-level clustering. The findings do not establish a causal mechanism or demonstrate transfer to broader motor competence. Prospective multi-school studies with random allocation at the class or instructional-subgroup level, prospective fidelity monitoring, blinded outcome assessment, and broader motor and process outcomes are required.

## Data Availability

The datasets presented in this article are not readily available because the data analysed in this study are derived from routine, school-based assessment records and are subject to institutional governance, ethical approval, and privacy-protection requirements. De-identified data are available from the corresponding author on reasonable request and with permission from the relevant institution and ethics committee. Requests to access the datasets should be directed to YL, 1039571063@qq.com.
